# Bibliometric Analysis of Neuroinflammation and Postoperative Cognitive Dysfunction

**DOI:** 10.1002/brb3.70271

**Published:** 2025-01-09

**Authors:** Zheping Chen, Zhenxiang Zuo, Yizheng Zhang, Guoliang Shan, Le Zhang, Moxuan Gong, Yuyang Ye, Yufeng Ma, Yanwu Jin

**Affiliations:** ^1^ Shanghai Key Laboratory of Anesthesiology and Brain Functional Modulation, Translational Research Institute of Brain and Brain‐Like Intelligence, Clinical Research Center for Anesthesiology and Perioperative Medicine, Department of Anesthesiology and Perioperative Medicine, Shanghai Fourth People's Hospital, School of Medicine Tongji University Shanghai People's Republic of China; ^2^ Department of Anesthesiology, the Second Hospital, Cheeloo College of Medicine Shandong University Jinan People's Republic of China; ^3^ Department of Gastroenterology, the Second Hospital, Cheeloo College of Medicine Shandong University Jinan People's Republic of China

**Keywords:** postoperative cognitive dysfunction, neuroinflammation, postoperative delirium, perioperative neurocognitive disorders, epigenetic modifications

## Abstract

**Background:**

The occurrence and development of postoperative cognitive dysfunction (POCD) are closely linked to neuroinflammation. This bibliometric analysis aims to provide novel insights into the research trajectory, key research topics, and potential future development trends in the field of neuroinflammation‐induced POCD.

**Methods:**

The Web of Science Core Collection (WoSCC) database was searched to identify publications from 2012 to 2023 on neuroinflammation‐induced POCD. Bibliometric analysis, involving both statistical and visual analyses, was conducted using CiteSpace, VOSviewer, and the R software.

**Results:**

Research on neuroinflammation‐induced POCD has exhibited an increasing trend over the past 12 years. China had the highest number of publications, Nanjing Medical University had the most collaboration with other institutions, Zhiyi Zuo was the most published author, and the *Journal of Neuroinflammation* served as the primary publication in the field of neuroinflammation‐induced POCD. The most frequent keyword was POCD. Keyword clustering analysis indicated that the predominant cluster is dexmedetomidine. Burst detection revealed that postoperative delirium (POD), perioperative neurocognitive disorders (PND), apoptosis, and epigenetic modifications were the future research trends.

**Conclusions:**

Our analysis identified the following key research areas associated with neuroinflammation‐induced POCD: anesthesia, surgery, dexmedetomidine, NLRP3 inflammasome, and mechanism of neuroinflammation‐induced POCD. The potential future research topics comprise POD, PND, apoptosis, and epigenetic modifications.

## Graphical abstract

Bibliometrics provides quantitative methods for investigating existing literature in a specific field. Our analysis identified the following key research areas associated with neuroinflammation‐induced POCD: anesthesia, surgery, dexmedetomidine, NLRP3 inflammasome, and mechanism of neuroinflammation‐induced POCD. The potential future research topics comprise POD, PND, apoptosis, and epigenetic modifications.

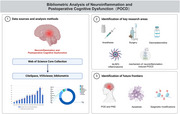

## 1 Introduction

Postoperative cognitive dysfunction (POCD) is a prevalent complication affecting the central nervous system (CNS) that results from anesthesia and surgical procedures (Dilmen et al. 2024; Yang et al. 2022). Clinically, POCD presents with cognitive impairments that affect vital areas such as memory, executive function, attention, language, and visual‐spatial abilities (Alam et al. 2018; Hanning 2005; Moller et al. 1998). These impairments typically occur after surgical procedures, with their impacts lasting from weeks to months. The concept of POCD has gradually evolved over the last decade, culminating into the introduction of perioperative neurocognitive dysfunction (PND) in 2018 based on the clinical terminology recommendations of the Diagnostic and Statistical Manual of Mental Disorders, Fifth Edition (DSM‐5). Perioperative neurocognitive dysfunction encompasses postoperative delirium (POD), delayed neurocognitive recovery (DNR), and POCD (Evered et al. 2018). Notably, POCD significantly impacts patient's quality of life and initial prognosis, contributing to increased postoperative morbidity and mortality; it has garnered extensive attention in recent years. Risk factors associated with POCD include advanced age, presence of comorbidities, extended surgical duration, and postoperative management practices (Evered and Silbert 2018; Khaled et al. 2024; Kubota et al. 2018). Consequently, POCD has emerged as a crucial area of research within the field of anesthesia, presenting an issue that demands urgent resolution (Liu et al. 2022; Yang et al. 2022).

Extensive research has been conducted on the pathophysiological mechanism of POCD, with previous studies investigating various factors including genes, molecules, neurons, and neural circuits. Results from these studies have revealed that various pathogenetic mechanisms of POCD ultimately converge on a common pathway known as neuroinflammation (Battaglia, Di Fazio, Mazzà, Tamietto, and Avenanti 2024, and Thayer 2024; Chen et al. 2019; Granger and Barnett 2021; Tanaka et al. 2024; Tanaka and Vécsei 2024; Yang et al. 2022). Neuroinflammation potentially impacts specific brain regions and neural circuits associated with cognitive function, such as the hippocampus and prefrontal cortex (mPFC), resulting in the occurrence of POCD (Battaglia, Harrison, and Fullana 2022; Ling et al. 2015). Imbalances in the neural circuits among key brain regions, such as the prelimbic cortex (PL)‐basolateral amygdala (BLA), infralimbic cortex (IL)‐basomedial amygdala (BMA), and lateral habenula (LHb)‐ventral tegmental area (VTA), contribute to the deterioration of cognitive function and decline in learning memory, which lead to POCD (Sun et al. 2023; Xin et al. 2022). Furthermore, activation of microglia triggers neuroinflammation, which plays a significant role in the occurrence and development of POCD. Microglia are resident immune cells within the CNS and are essential enabling the crosstalk between neuroinflammation and processes such as neurodegeneration, neuroapoptosis, synaptic injury, and disruption of blood‐brain barrier (BBB) (Hu et al. 2018; Ransohoff 2016; Skvarc et al. 2018). Neuroinflammation, particularly involving the activation of microglia, has been significantly implicated in the pathophysiological mechanisms of POCD among the elderly.

Due to the growing issue of global aging, the prevalence of POCD among surgical patients has been escalating, making POCD a significant concern in current medical research and practice. An existing bibliometric analysis indicates that neuroinflammation plays a significant role in the investigations on POCD (Chen et al. 2020). However, identifying research hotspots and emerging trends in neuroinflammation‐induced POCD remains a challenge. Bibliometrics provides quantitative methods for investigating existing literature in a specific field; consequently, it is the most used literature analysis tool (Chen and Song 2019; Li et al. 2015). In this study, we conducted a comprehensive analysis of neuroinflammation‐induced POCD studies published within the past 12 years, thereby developing a visual model to elucidate the current research status and hot spots. This study provides a vital foundation to guide future developments and investigations into mechanism of neuroinflammation in POCD.

## 2 Data Sources and Analysis Methods

### 2.1 Data Sources

The Web of Science Core Collection (WoSCC) was selected as the main database for conducting bibliometric analysis. This database was identified based on various factors. First, WoSCC is rich with diverse academic resources encompassing various fields and disciplines. Notably, WoSCC is extensively used in bibliometric analysis due to its systematic, authoritative, and comprehensive nature, thereby rendering it an ideal data source for our research (Peng et al. 2022; Zhang et al. 2024). Second, data from WoSCC are compatible with numerous bibliometric software tools, minimizing the risk associated with data corruption or loss caused through data conversion; thereby maintaining the integrity and accuracy of the analysis (Wang et al. 2024). Finally, Bradford's Law can be applied to data from WoSCC, enabling the identification of core publications and journals.

### 2.2 Search Strategy

**FIGURE 1:**
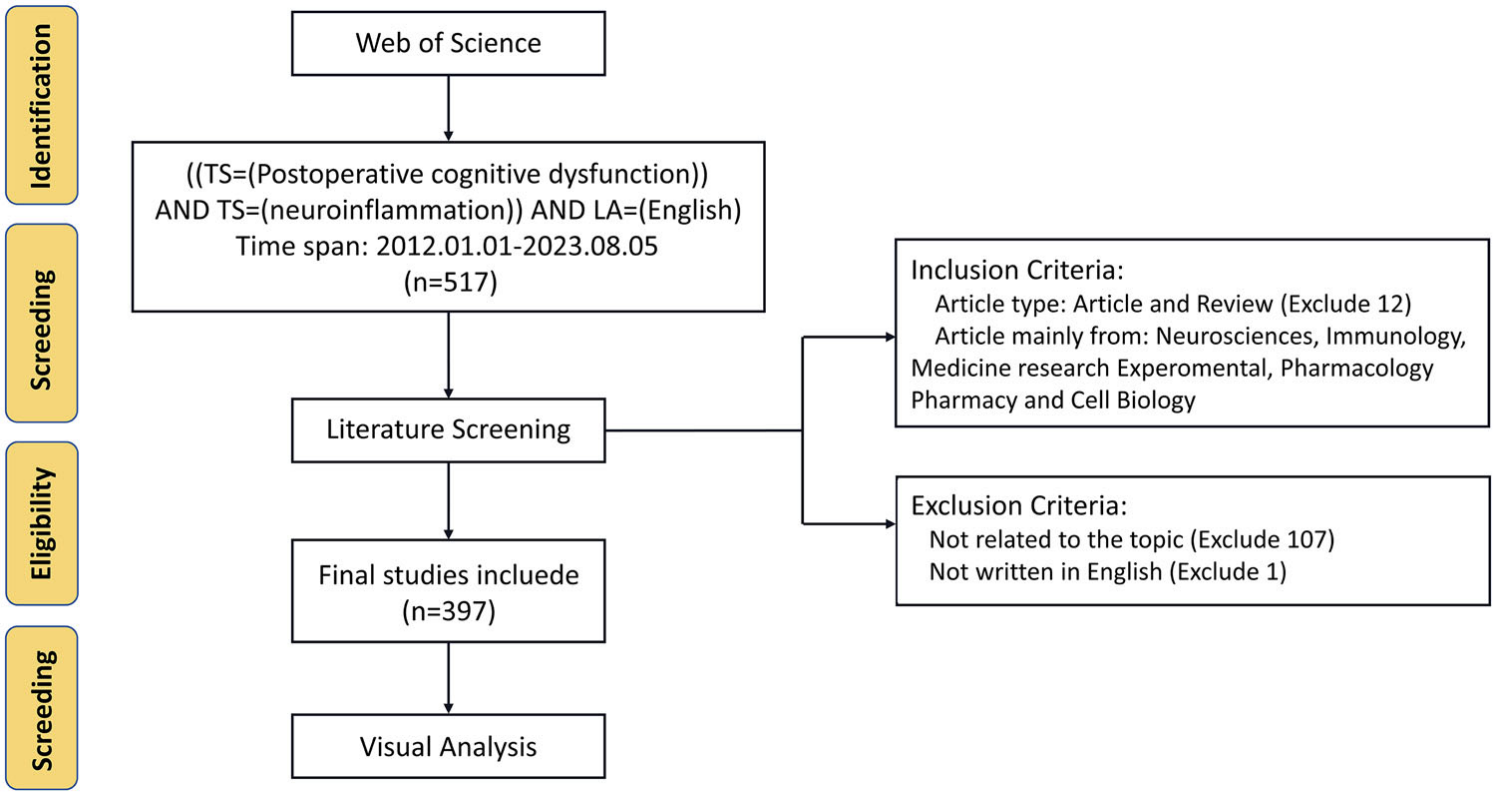
PRISMA study flowchart.

The search strategy applied was as follows: [Postoperative cognitive dysfunction] AND [neuroinflammation], covering the period between 2012 and 2023. Search results including abstracts, editorials, protocols, letters, retracted publications, bibliometric analyses, and expressions of concern were excluded. The exclusion criteria were as follows: (1) POCD is not the central focus of the publication; (2) non‐English publications; (3) unavailability of full text; and (4) duplicate publications. Figure 1 shows a comprehensive depiction of the entire retrieval and screening procedure.

### 2.3 Data Extraction and Cleansing

Data download were completed by August 6, 2023, while the entire literature search was conducted on August 5, 2023 to mitigate potential bias due to daily database updates. The data search and cleaning processes were conducted by two sets of trained and independent data reviewers. In cases of disagreements regarding inclusion, a discussion involving a third researcher was used to achieve consensus, thereby resolving such cases. A total of 397 articles were ultimately identified (Figure 1). To ensure the quality of the research, we merged keywords and duplicate authors, including synonyms, aliases, plurals, and case of names.

The screened publications were exported in both Refworks and plain text formats. The data collected included “full records and cited references” and were stored in the downloaded format as (_*.txt). The format conversion of the retrieved publications was performed using the Data Import/Export functionality in VOSviewer (1.6.19), CiteSpace software (6.3.R3), and R (4.3.1) “bibliometrix” (https://www.bibliometrix.org).

### 2.4 Bibliometric Visualization

The CiteSpace (6.3.R3), a visualization software developed by Professor Chaomei Chen using the Java platform, can be used to effectively identify prominent scientific institutions, authors, journals, references, and keywords. This software was used to conduct cluster visualization, timeline visualization, volcano charts, and burst detection (Chen 2004; Chen and Song 2019).

The “Bibliometrix” package (https://www.bibliometrix.org) in R software is a powerful and specialized tool for conducting quantitative analysis in the bibliometrics and scientometrics fields (Arruda et al. 2022). We utilized this package to plot tri‐field maps, research topic evolution paths, and analyze trends in countries, authors, journals, and citations.

The VOSviewer (1.6.19) (https://www.vosviewer.com/) was used to construct bibliometric maps illustrating the density distributions of perspectives of the authors (van Eck and Waltman 2010). The visual network analysis of collaborative efforts between authors was generated using VOSviewer and SCImago Graphica (1.0.36) (https://graphica.app/).

### 2.5 Data Analysis

The visualization map generated using CiteSpace contained various nodes. Each node represents an entity such as an institution, author, journal, reference, or keyword. The size of the nodes indicates the frequency of occurrence or citation, while the colors of the nodes represented varying years (Sabé et al. 2023). The centrality of each node was represented by the outer purple ring in the CiteSpace's visualization maps, with thicker rings indicating higher degrees of centrality. Nodes with a high centrality value of > 0.1 are typically considered pivotal or critical points within a field (Wang et al. 2023).

For cluster analysis, the log‐likelihood ratio (LLR) was used to calculate the modularity (*Q* value) and mean silhouette score (*S* value). These values were used to evaluate the overall structural features of the network, with the quality of the cluster considered satisfactory when *Q* > 0.3 and *S* > 0.5 (Rousseeuw 1987; Sabé et al. 2023).

In the burst test, the presence of a red ring indicates the occurrence of a burst within that year, whereas the color of the node indicates a particular time interval. The sizes of the nodes represent the number of projects, whereas size of the lines correspond to the degree of collaboration and occurrence (Xu et al. 2021).

The visual analysis of authors was conducted using Price's Law formula (Mp = 0.749*$\sqrt{\mathrm{Npmax}}$; where Npmax indicates the maximum number of papers published by the most productive authors). Authors with publications that exceeded the calculated value, Mp, were considered core authors.

## 3 Results

### 3.1 Bibliometric Analysis of Publication Outputs

**FIGURE 2:**
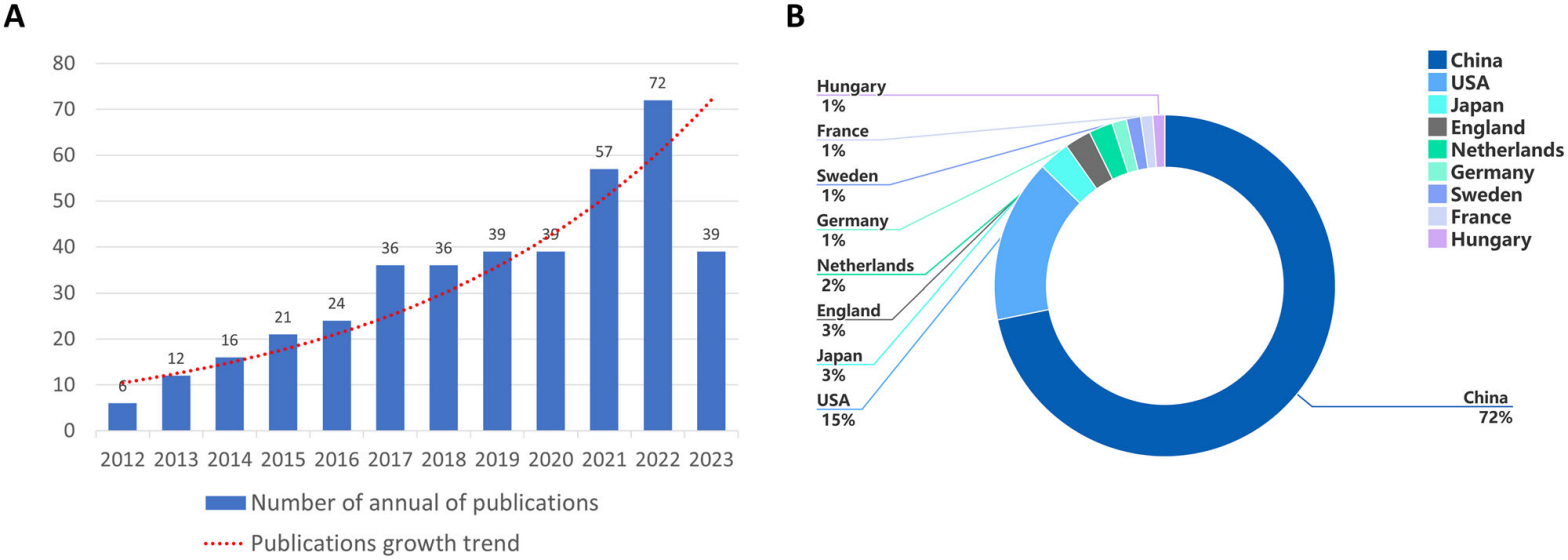
Bibliometric analysis of publication output. (A) The number of annual publications; (B) The distribution of publications by countries/regions.

The annual publication volume served as a crucial metric indicating the level of attention devoted to a specific research field. The visual representation of the time distribution of papers published in the field of neuroinflammation‐induced POCD is shown in Figure 2. A total of 397 papers were included in the analysis. Overall, the number of publications in the field of neuroinflammation‐induced POCD increased 12‐fold, from 6 in 2012 to 72 in 2022. In 2021 and 2022, the number of published papers was above 50, indicating a growing interest in the field of neuroinflammation‐induced POCD. Notably, 52.1% of the 72 publications were released within the past 4 years, as depicted in Figure 2A. Among the 25 countries/regions involved in the study, China contributed 316 publications, accounting for 79.6% of all publications, as shown in Figure 2B.

### 3.2 Analysis of Country/Region Cooperation and Volume of Articles Issued

The top five productive countries were China (*n* = 316), the USA (*n* = 68), Japan (*n* = 13), England (*n* = 11), and Netherlands (*n* = 10) (Table 1). Despite China exhibiting the highest number of publications, its average citation count is significantly lower compared to that of the USA and England. Conversely, while Netherlands recorded the fewer publications, it has the highest average number of citations (Table 1).

**TABLE 1:** Publication number, centrality, and citations by country/region.

Rank	Country/Region	Publications	Citations	Average citations	Centrality
1	China	316	5888	18.63	0.10
2	USA	68	2243	32.99	0.54
3	Japan	13	212	16.31	0
4	England	11	368	33.45	0.28
5	Netherlands	10	667	66.70	0
6	Sweden	6	357	59.50	0.28
7	Germany	6	98	16.33	0.10
8	France	5	253	50.60	0.10
9	Hungary	5	133	26.60	0
10	Italy	4	191	47.75	0

**FIGURE 3:**
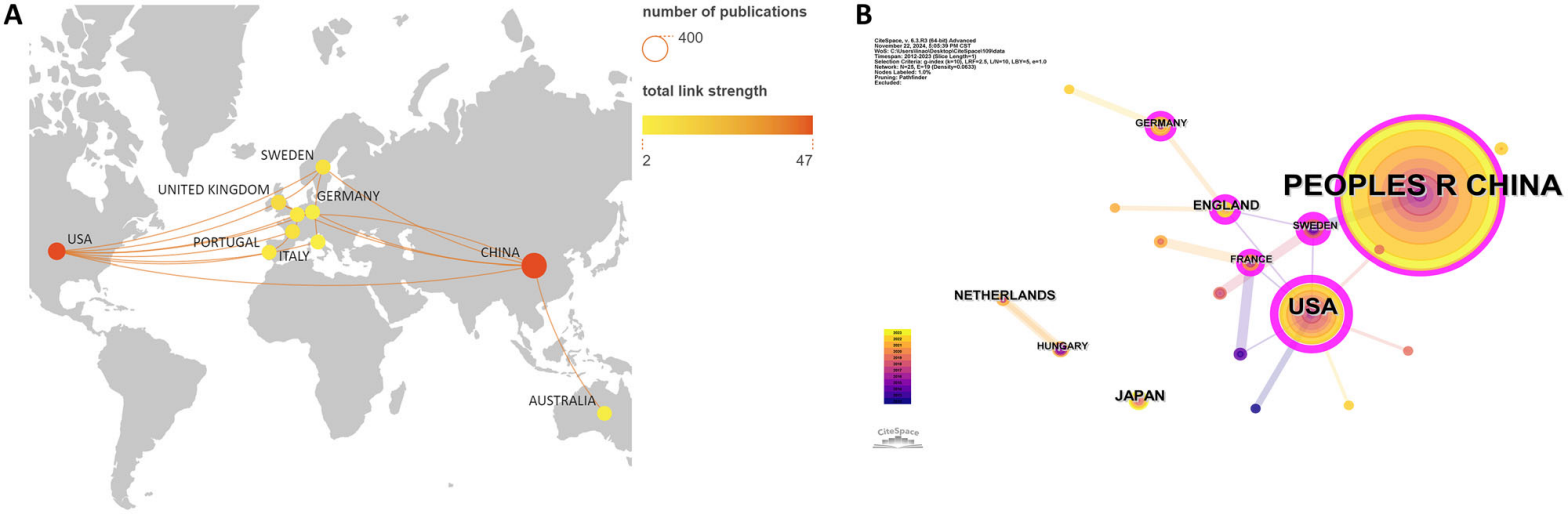
Cooperation between countries/regions on neuroinflammation‐induced POCD. (A) Geography map of national cooperative. The size of the circles shows the number of publications by country/region. There is a connection between countries/regions, implying that there is cooperation between the two countries/regions. The same color of the wires means that these countries/regions form academic groups; (B) The cooperation networks between different countries/regions using CiteSpace.

Geographical map of national cooperation (Figure 3A) shows that countries/regions there is limited cooperation among the entities involved. This observation highlights the need for intensified cross‐regional and transnational cooperation. Additionally, CiteSpace was used to conduct a collaborative national network knowledge mapping research of the articles, with the results revealing that numerous nations have contributed to the field of neuroinflammation‐induced POCD (Figure 3B). However, there is uneven distribution of the number of publications by various nation; with the majority of papers are written by the top ranked countries. Centrality assessments revealed that although China leads in publications, the USA has a significantly greater influence (centrality = 0.54), followed by England (centrality = 0.28), and Sweden (centrality = 0.28) (Figure 3B).

**FIGURE 4:**
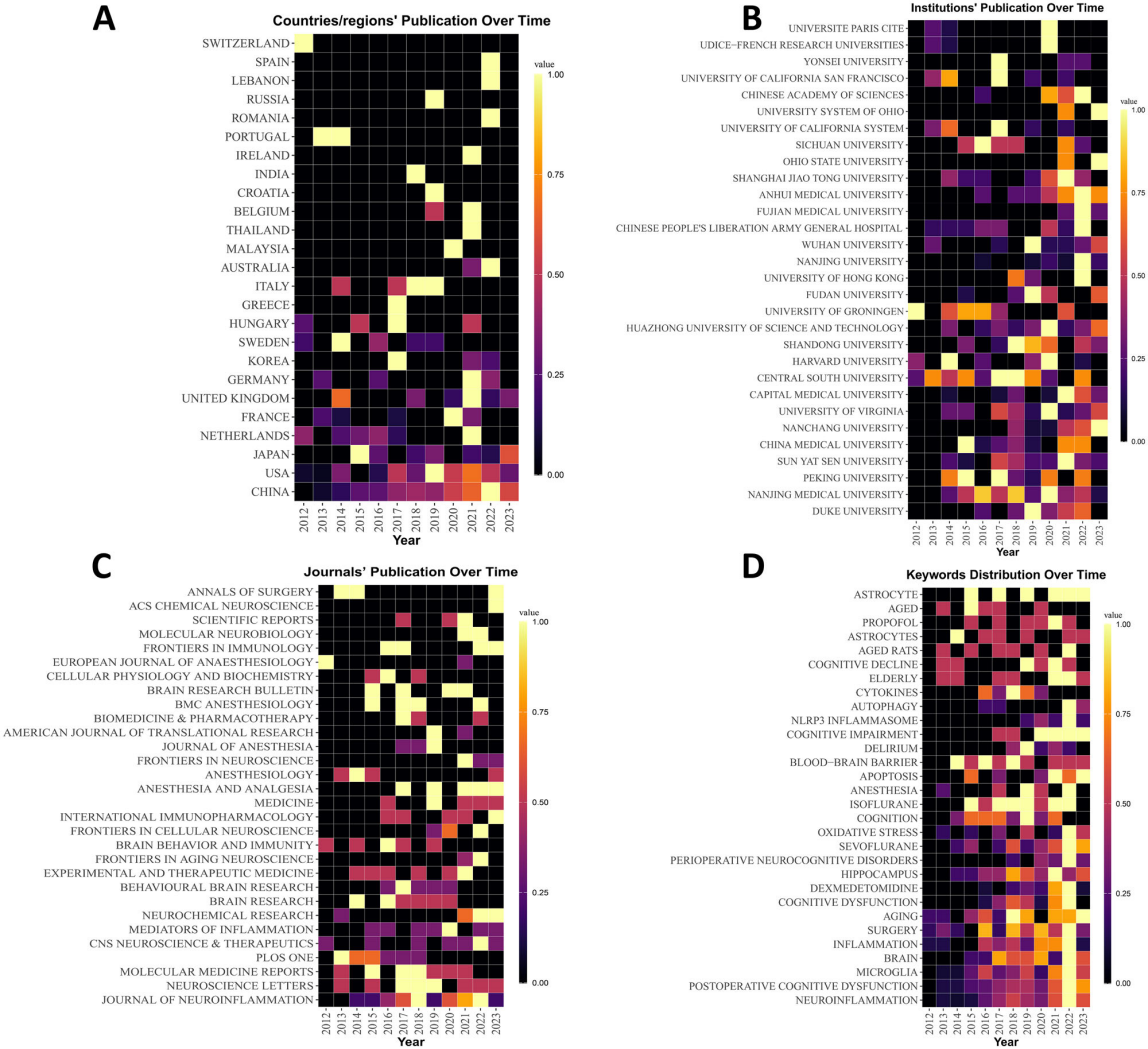
Annual fluctuations in academic output from 2012 to 2023. (A) high‐productivity countries/regions (top 25); (B) high‐productivity institutions (top 30); (C) high‐productivity journals (top 30); (D) high‐frequency keywords (top 30).

The annual publication trends among the top 25 countries/regions, depicted in Figure 4A, suggest that majority of these countries/regions experienced a peak in publication output from 2020 to 2022. These observations indicate that neuroinflammation‐induced POCD has garnered significant attention in the past decade.

### 3.3 Analysis of the Collaboration of Institutions and Volume of Publications

**FIGURE 5:**
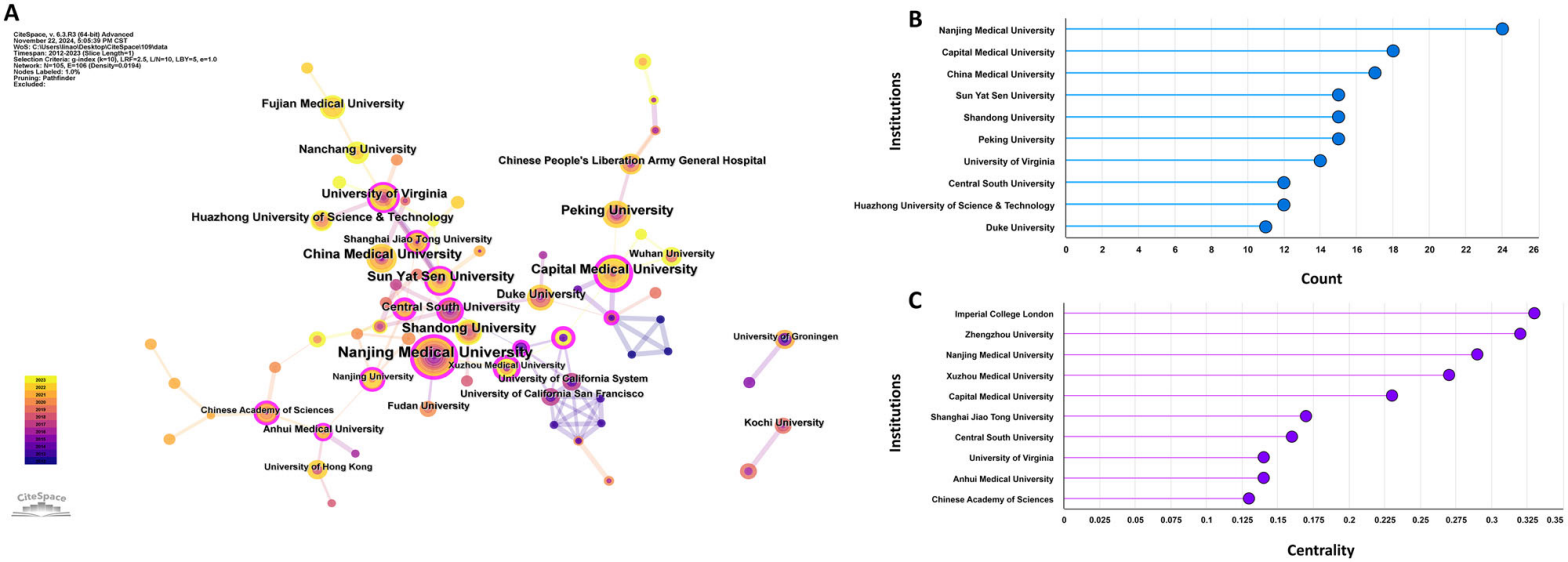
Cooperation between institutions on neuroinflammation‐induced POCD using CiteSpace. (A) The cooperation networks between different institutions; (B) The top 10 institutions in count; (C) The top 10 institutions in centrality.

A total of 105 institutions were included in all publications. Subsequently, we used CiteSpace to conduct a visual analysis of the interconnections between these institutions, with the cooperation network visualizations map of the institutions presented in Figure 5A. The Nanjing Medical University (*n* = 24) collaborated most with other institutions (Figure 5B). Network visualizations revealed that the top three institutions in terms of centrality were the Imperial College London (centrality =  0.33), the Zhengzhou University (centrality = 0.32), and Nanjing Medical University (centrality = 0.29) (Figure 5C). These institutions act as bridges within the network and are actively involved in cooperative research. These institutions reached peaked in their publication volume from 2019 to 2022 (Figure 4B).

### 3.4 Analysis of the Higher‐impact Journals

**FIGURE 6:**
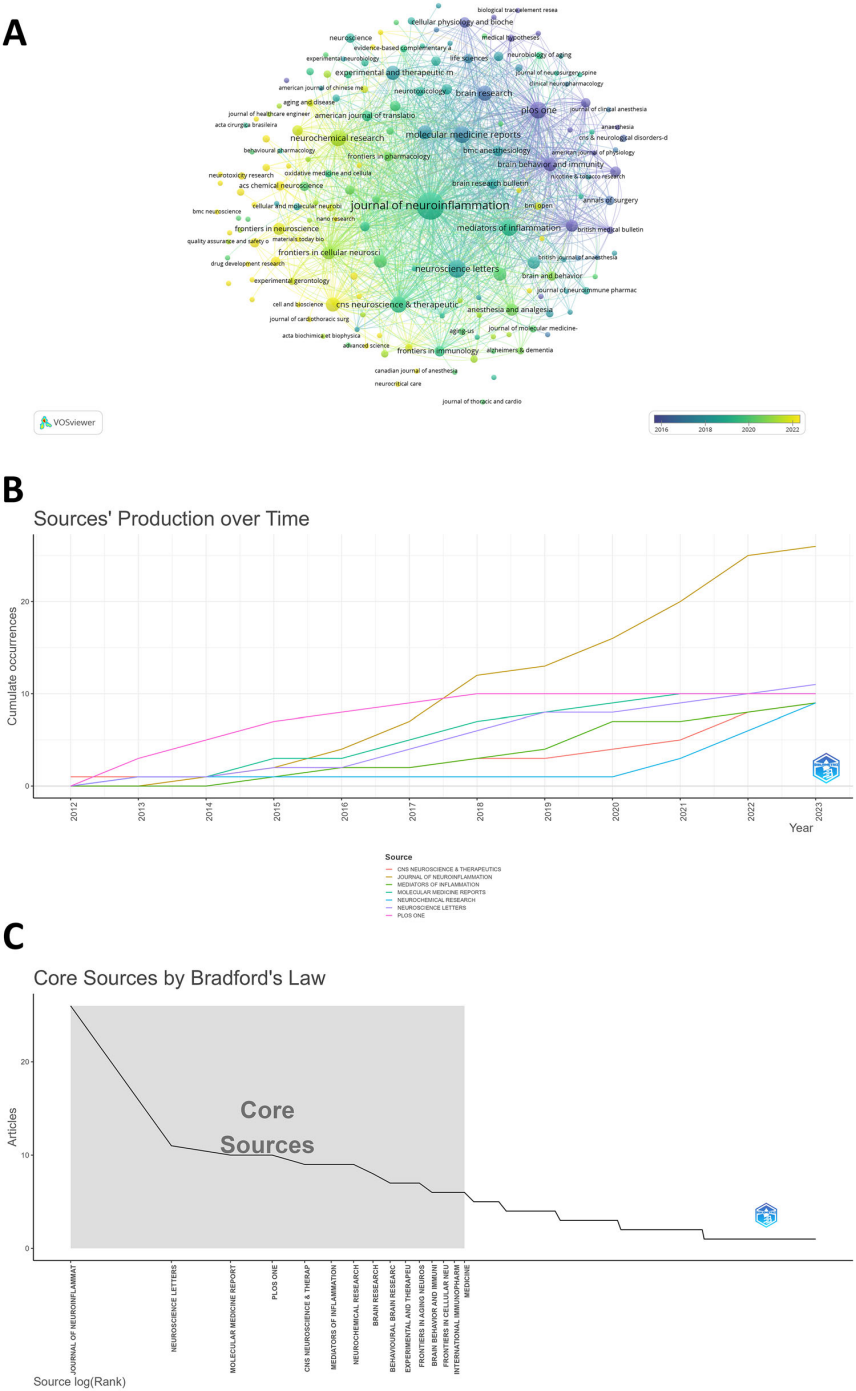
Journals analysis on neuroinflammation‐induced POCD using VOSviewer. (A) The overlay visualization map of journals from 2012 to 2023. Node size and color represents total number of citations and a single time slice, respectively; (B) Journal output trends within the top 7 from 2012 to 2023. (C) Core journals by Bradford's Law.

A total of 168 journals published articles on POCD neuroinflammation. The collaboration mapping of journals, constructed using VOSviewer and shown in Figure 6A, revealed that the *Journal of Neuroinflammation* (IF = 9.30, Q1) had the highest volume of citations relevant published literature.

*Journal of Neuroinflammation* has published 26 articles, with a total of 1,011 citations, focusing on the occurrence and spread of neuroinflammation, interaction and immune response between microglia, and various immune cells and neurons. These factors have extensively been explored and often regarded as core mechanisms of neurodegenerative diseases. The earliest research in this field has been published in renowned anesthesiology and surgery journals, such as *British Journal of Anaesthesia*, *Journal of Clinical Anesthesia*, and *Annals of Surgery*. Currently, there has been a shift of focus towards vital areas, including neuroscience, molecular medicine, pharmacology, and translational medicine, as shown in Figure 6A.

The number of neuroinflammation‐induced POCD studies published in journals has rapidly increased since 2015, peaking between 2019–2023 (Figures 4C and 6B). Using Bradford's Law, the 168 journals were divided into three zones (1–3), resulting in 15 core source journals being assigned to zone 1 (Figure 6C) (Tables 2 and 3).

**TABLE 2:** According to Bradford's Law, the 168 journals were classified into zones 1–3.

Zone	Number of journals	Number of publications	Percentage
1	15	137	34.51%
2	42	129	32.49%
3	111	131	33.00%
Total	168	397	100%

**TABLE 3:** Fifteen core source journals in zone 1 according to Bradford's Law.

Rank	Journal	Count
1	Journal of Neuroinflammation	26
2	Neuroscience Letters	11
3	Molecular Medicine Reports	10
4	Plos One	10
5	CNS Neuroscience & Therapeutics	9
6	Mediators of Inflammation	9
7	Neurochemical Research	9
8	Brain Research	8
9	Behavioural Brain Research	7
10	Experimental and Therapeutic Medicine	7
11	Frontiers in Aging Neuroscience	7
12	Brain Behavior and Immunity	6
13	Frontiers in Cellular Neuroscience	6
14	International Immunopharmacology	6
15	Medicine	6

### 3.5 Analysis of the Most Influential Authors and Co‐Cited Authors

**FIGURE 7:**
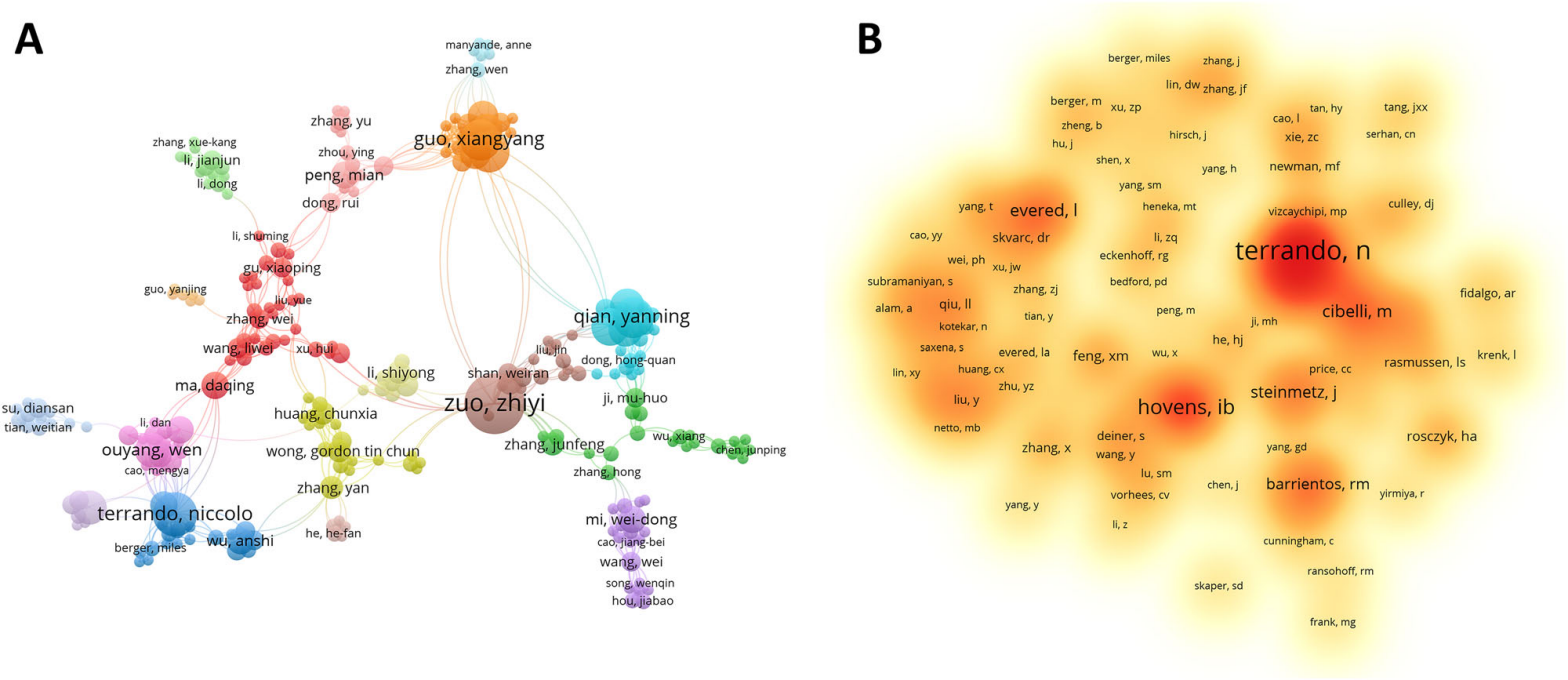
Authors analysis on neuroinflammation‐induced POCD. (A) The cooperation networks between different authors using VOSviewer; (B) The density visualization map of co‐cited authors based on VOSviewer. The density of color indicates how many publications for authors.

Analysis of influential authors in publications facilitated the identification of authoritative authors and core research forces involved in the research on POCD neuroinflammation. Using VOSviewer, the maximum number of publications ($n_{\max}$) by a single author in this field is 16 (Figure 7A) and based on Price's Law, authors with over three publications were regarded as core authors on neuroinflammation‐induced POCD.

A total of 155 core authors were identified and Zhiyi Zuo from the University of Virginia emerged as the most prolific contributor, with 16 articles between 2012 and 2023 (Figure 7A) (Table 4). His articles garnered a citation count of 470, averaging 29.38 citations per article. Mervyn Maze from University of California‐San Francisco ranked top in citations and average citations (*n* = 75.88). The co‐citation visualization analysis (Figure 7B), revealed that Niccolò Terrando—a renowned expert in neuroinflammation studies—from Duke University Medical Center was the most co‐cited author with 357 citations. These three authors are prominent personalities within the field of anesthesiology.

**TABLE 4:** Top 10 authors ranked by the publications.

Rank	Author	Publications	Citations	Average citations
1	Zhiyi Zuo	16	470	29.38
2	Niccolò Terrando	11	560	50.91
3	Yanning Qian	10	474	47.40
4	Zhengqian Li	10	227	22.70
5	Xiangyang Guo	10	227	22.70
6	Jie Sun	9	372	41.33
7	Xuezhao Cao	9	127	14.11
8	Mervyn Maze	8	607	75.88
9	Hongquan Dong	8	445	55.63
10	Xiang Zhang	8	439	54.88

### 3.6 Analysis of Co‐Cited Journals

Analysis of co‐cited journals is vital in assessing emerging trends and identifying promising research prospects within a specific academic domain. The frequency at which a particular journal is cited indicates its impact as it demonstrates its recognition within a specific academic field. The highest number of citations was observed in the “*Journal of Neuroinflammation*” (*n* = 1011) and “*Brain Behavior and Immunity*” (*n* = 586) (Table 5).

**TABLE 5:** Top 10 co‐cited journals ranked by the citations.

Rank	Journals	Citations	JCR	IF (2023)
1	Journal of Neuroinflammation	1011	Q1	9.3
2	Brain Behavior and Immunity	586	Q1	8.8
3	Plos One	369	Q1	2.9
4	Anesthesiology	336	Q1	9.3
5	Behavioural Brain Research	265	Q2	2.6
6	CNS Neuroscience & Therapeutics	246	Q1	4.8
7	Brain Research	230	Q3	2.7
8	Anesthesia & Analgesia	206	Q1	4.6
9	Neuroscience Letters	203	Q3	2.5
10	Molecular Medicine Reports	195	Q2	3.4

**FIGURE 8:**
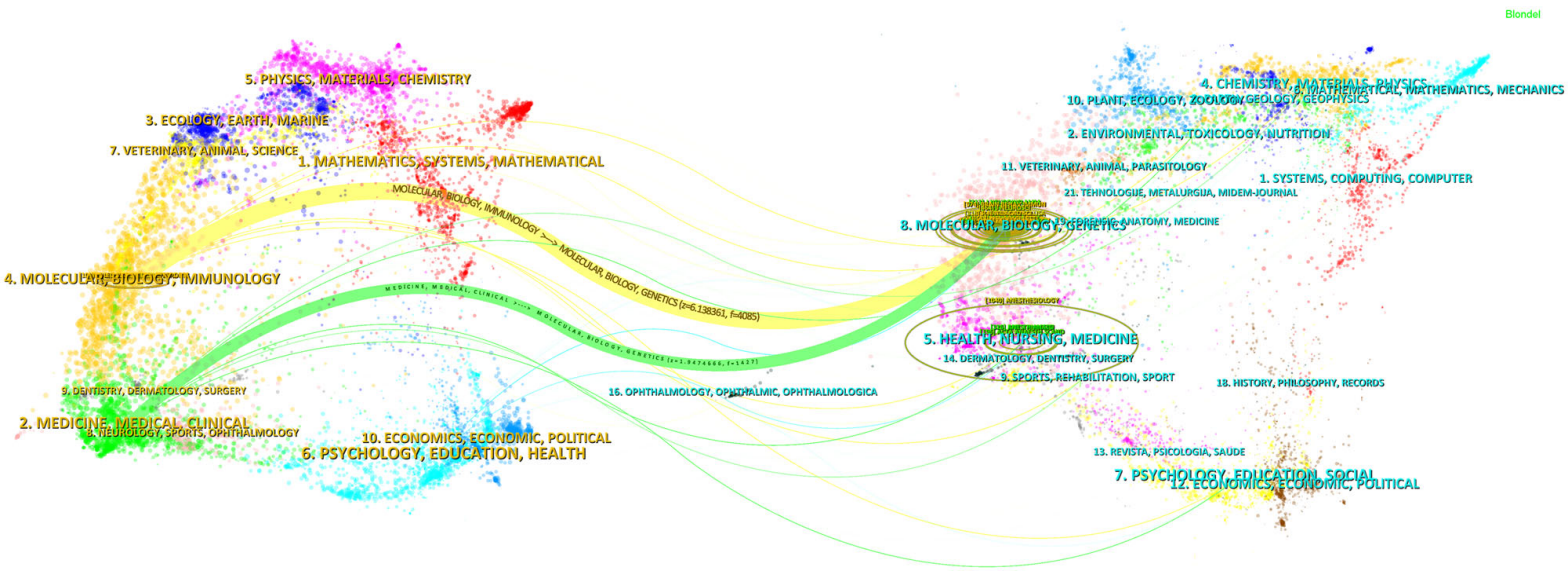
The dual‐map overlay of citing of citation relationship of articles on neuroinflammation‐induced POCD, with citing journal on the left, and the cited journal on the right. The colored path represented the citation relationship.

We constructed a dual‐map overlay of journals that depicted the relationships between citing and cited journals. The left side representing a group of citing journals, while the right side representing a group of cited journals (Figure 8). The results show that when the molecular/biology/immunology category served as the source journal, the molecular/biology/genetics category has the highest citation count in the field of neuroinflammation‐induced POCD (*Z*‐score = 6.138).

### 3.7 Analysis of Co‐Cited References

**FIGURE 9:**
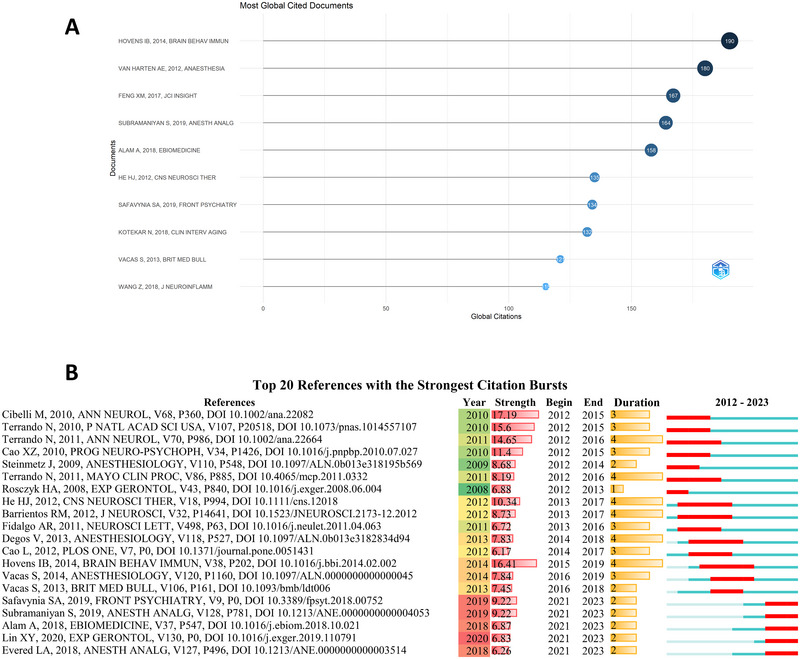
Cited references analysis on neuroinflammation‐induced POCD. (A) The top 10 co‐cited references in count using R; (B) The top 20 co‐cited references with the strongest citation bursts.

The most frequently co‐cited publication was by Hovens et al., published in 2014 in *Brain Behavior and Immunity*, aggregating 190 citations (Hovens et al. 2014). Hoven et al. investigated the temporal progression of neuroinflammation after surgery, alterations in the BDNF pathway, and their association with POCD. They observed that postoperative alterations in neuroinflammation, BDNF pathway, and neurogenesis are fundamental components in the underlying mechanism for POCD (Hovens et al. 2014) (Figure 9A).

The top 20 references with the strongest citation bursts are presented in Figure 9B. The blue and red line segments represent the timeline and duration, respectively. A rapid increase in citations to specific literature within a certain timeframe suggest emergence of a potential research trend. The top ranked article in burst strength (strength = 17.19) was the “Role of interleukin‐1beta in postoperative cognitive dysfunction”, authored by Mario Cibelli from Imperial College London and published in *Annals of Neurology* in 2010 (Figure 9B). Furthermore, high‐impact articles for clinical and animal experimentation are presented in Tables 6 and 7, respectively.

**TABLE 6:** Top 10 publications concerning clinical studies in ranked by the citations.

Rank	References	Publication year
1	Postoperative cognitive dysfunction after inhalational anesthesia in elderly patients undergoing major surgery: the influence of anesthetic technique, cerebral injury and systemic inflammation	2015
2	Perioperative cerebrospinal fluid and plasma inflammatory markers after orthopedic surgery	2016
3	Effect of propofol, sevoflurane, and isoflurane on postoperative cognitive dysfunction following laparoscopic cholecystectomy in elderly patients: a randomized controlled trial	2017
4	Perioperative inflammatory response and protein S‐100β concentrations—relationship with post‐operative cognitive dysfunction in elderly patients	2012
5	BIS‐guided deep anesthesia decreases short‐term postoperative cognitive dysfunction and peripheral inflammation in elderly patients undergoing abdominal surgery	2019
6	Parecoxib prevents early postoperative cognitive dysfunction in elderly patients undergoing total knee arthroplasty: a double‐blind, randomized clinical consort study	2016
7	Neuroprotective effects of intravenous lidocaine on early postoperative cognitive dysfunction in elderly patients following spine surgery	2015
8	Influence of postoperative analgesia on systemic inflammatory response and postoperative cognitive dysfunction after femoral fractures surgery: a randomized controlled trial	2019
9	Effects of methylprednisolone on blood‐brain barrier and cerebral inflammation in cardiac surgery‐a randomized trial	2018
10	Recovery of postoperative cognitive function in elderly patients after a long duration of desflurane anesthesia: a pilot study	2015

**TABLE 7:** Top 10 publications concerning animal studies in ranked by the citations.

Rank	References	Publication year
1	Postoperative cognitive dysfunction: Involvement of neuroinflammation and neuronal functioning	2014
2	Microglia mediate postoperative hippocampal inflammation and cognitive decline in mice	2017
3	Surgery upregulates high mobility group box‐1 and disrupts the blood‐brain barrier causing cognitive dysfunction in aged rats	2012
4	Critical role of NLRP3‐caspase‐1 pathway in age‐dependent isoflurane‐induced microglial inflammatory response and cognitive impairment	2018
5	Dysregulation of BDNF/TrkB signaling mediated by NMDAR/Ca^2+^/calpain might contribute to postoperative cognitive dysfunction in aging mice	2020
6	Activated brain mast cells contribute to postoperative cognitive dysfunction by evoking microglia activation and neuronal apoptosis	2016
7	Depletion of bone marrow‐derived macrophages perturbs the innate immune response to surgery and reduces postoperative memory dysfunction	2013
8	MicroRNA‐146a protects against cognitive decline induced by surgical trauma by suppressing hippocampal neuroinflammation in mice	2019
9	Aspirin‐triggered resolving D1 prevents surgery‐induced cognitive decline	2013
10	High‐mobility group box 1 protein initiates postoperative cognitive decline by engaging bone marrow‐derived macrophages	2014

### 3.8 Research Themes

**FIGURE 10:**
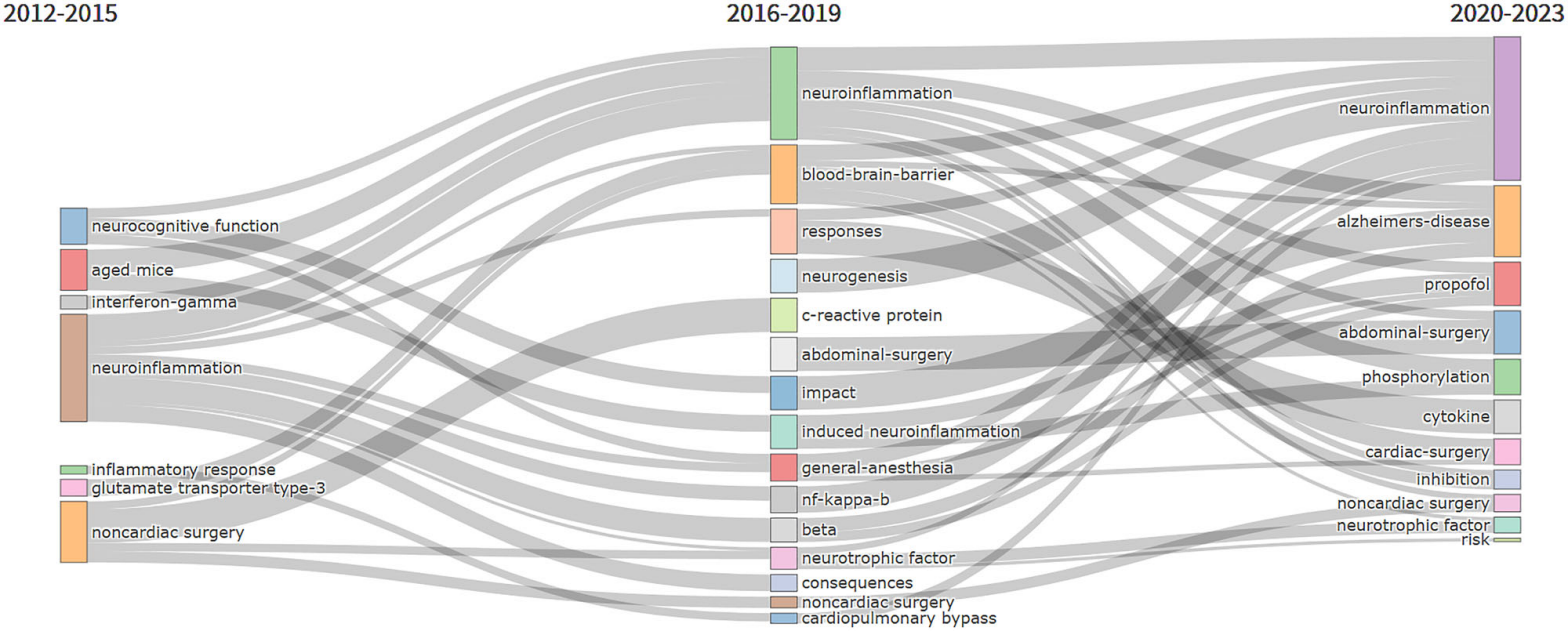
R bibliometrics‐thematic evolution tool traces the progression of research themes in the realm of neuroinflammation‐induced POCD, spanning from 2012 to 2023. The time segmentation points were established in 2015 and 2019 using R.

To provide a comprehensive analysis of the dynamic shifts in research themes, bibliometrics‐thematic evolution tool in R software was used to examine the trajectory of research themes among neuroinflammation‐induced POCD studies from 2012 to 2023 (Figure 10).

It has been established that the early investigations into neuroinflammation‐induced POCD were confined to exploring potential inflammatory responses and associated mechanisms in model animals, such as aged mice. Over time, in‐depth inquiries have been conducted, including those related to the BBB and neuroprotective factors. Recently, clinical studies have also examined the risk of neuroinflammation‐induced POCD in patients undergoing cardiac, non‐cardiac, and major abdominal surgical procedures, as well as those with Alzheimer's disease. This indicates that research on neuroinflammation‐induced POCD, integrating both clinical and basic aspects, has emerged as the primary focus and prominent topic of interest.

### 3.9 Analysis of Keywords

#### 3.9.1 Keywords Co‐Occurrence

**FIGURE 11:**
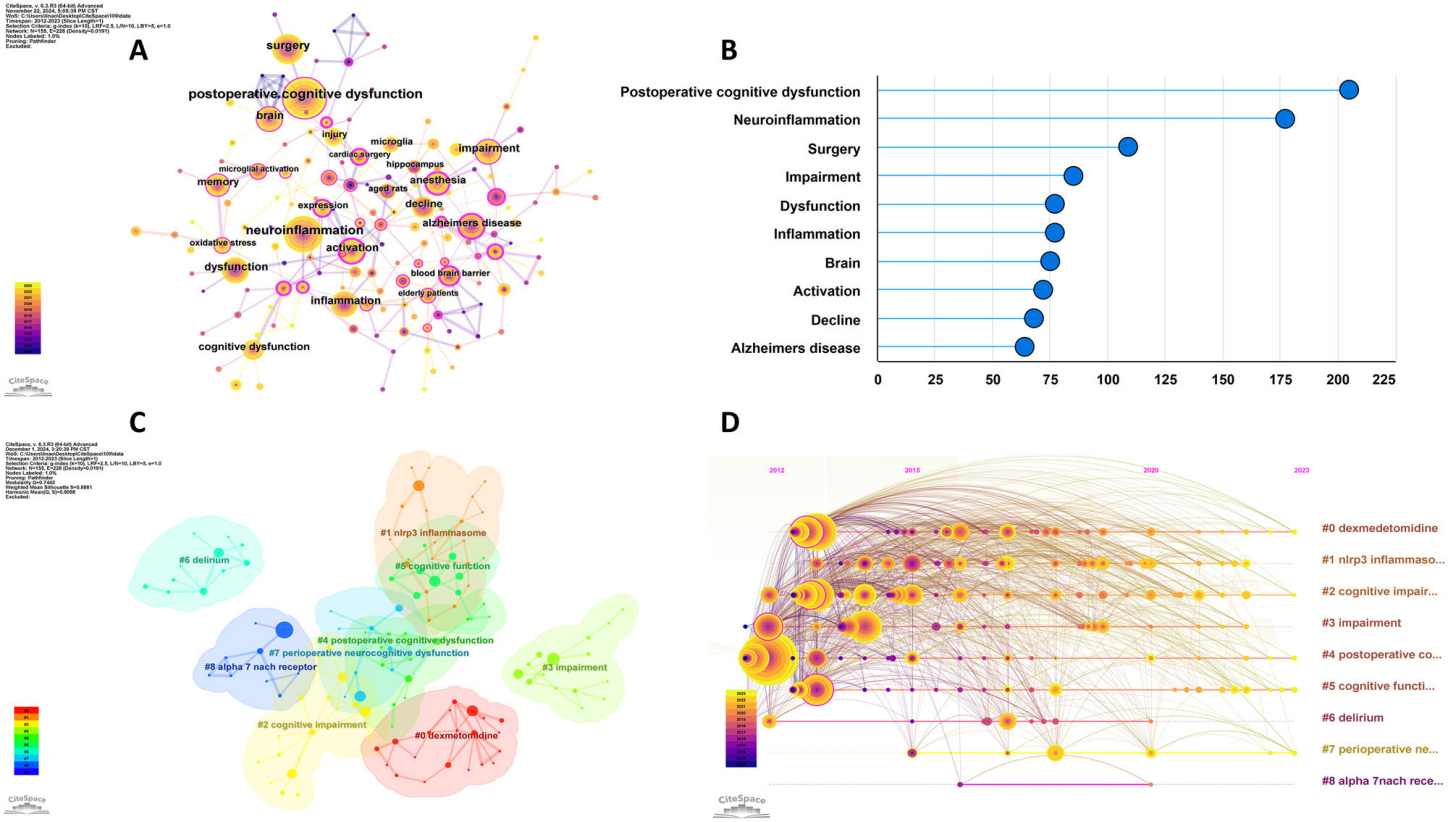
Keywords analysis on neuroinflammation‐induced POCD. (A) The network map of the co‐occurrence keywords using CiteSpace; (B) The top 10 keywords in count; (C) Clusters of keywords using CiteSpace; (D) Timeline view of keywords analysis using CiteSpace.

Analysis of keywords co‐occurrence in numerous literary works effectively facilitates the categorization of high‐frequency terms and evaluation of the intensity of their associations. This approach enables us to uncover the internal structure of an academic field, along with its research frontiers. The co‐occurrence network of keywords is depicted in Figure 11A. Expectedly, “POCD” (*n* = 205) emerged as the most frequent keyword in co‐occurrence analysis, with a significant focus directed to terms such as “anesthesia”, “microglia activation”, and “elderly patients” (Figure 11A,B). The top 25 keywords in terms of frequency reached a peak in research activity between 2020 and 2023 (Figure 4D).

#### 3.9.2 Clusters of Keywords

Cluster analysis was used to provide a deeper understanding of the fundamental knowledge framework in the field of neuroinflammation‐induced POCD. Data were classified according to their similarity using CiteSpace based on the LLR. The results revealed that the keywords could be divided into nine main clusters (*Q* value = 0.7442, *S* value = 0.8881) (Figure 11C): #0 dexmetomidine, #1 NLRP3 inflammasome, #2 cognitive impairment, #3 impairment, #4 postoperative cognitive dysfunction, #5 cognitive function, #6 delirium, #7 perioperative neurocognitive dysfunction, and #8 α7‐nAChR.

#### 3.9.3 Timeline of Keywords

We used CiteSpace to create a timeline diagram spanning 2012–2023 (Figure 11D). This diagram enabled for a deeper understanding of current research themes and in anticipating future directions in neuroinflammation‐induced POCD. The temporal occurrence of nodes is depicted by their position, while the frequency of their appearance is indicated by their size; these highlight areas of significant research interest. A total of nine clusters were identified, with their ranking determined by the volume of literature contained (Figure 11D).

#### 3.9.4 Keywords Bursts

**FIGURE 12:**
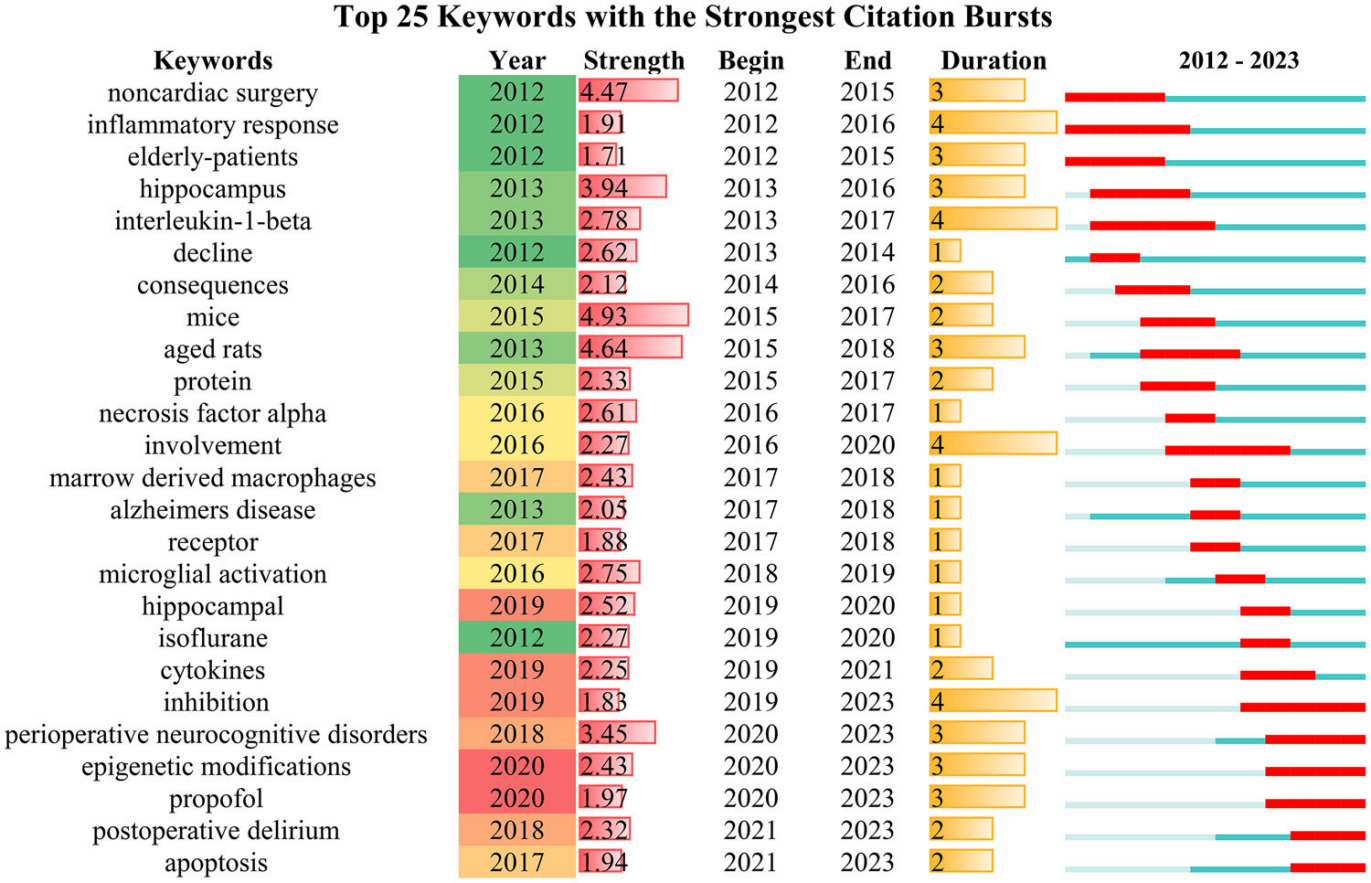
The top 25 keywords with the strongest citation bursts.

We used CiteSpace to identify the burst keywords in research hotspots. The burst map effectively illustrated that there was a temporal increase in keywords usage, enabling the assessment of research directions and attention levels during specific periods (Chen 2006). The blue line, representing the timeline, is marked with red grids to indicate the beginning and ending year; it also indicates the duration of a citation burst. As shown in Figure 12, “mice” ranked first with the highest burst strength (strength = 4.93), followed by “aged rats” (strength = 4.64), “noncardiac surgery” (strength = 4.47) and “hippocampus” (strength = 3.94). Currently, research hotspots include “apoptosis” and “postoperative delirium” (POD), “perioperative neurocognitive disorders” (PND) and “epigenetic modifications”. These topics have garnered significant attention and could serve as future potential research trends and hotspots in the field of neuroinflammation‐induced POCD.

## 4 Discussion

Bibliometrics methods were used to visually analyze a total of 397 neuroinflammation‐induced POCD publications from 2012 to 2023 using Bibliometrix in R, CiteSpace, and VOSviewer.

### 4.1 Diagnosis of POCD

The diagnosis of POCD was traditionally based on the symptoms of cognitive decline. However, in 2018 according to the standards outlined in the DSM‐5, the term was changed to PND. Notably, PND encompasses a broader range, encompassing POD within 7 days post‐surgery, DNR within 30 days, and POCD within 12 months post‐surgery (Kong, Xu, and Wang 2022; Migirov, Chahar, and Maheshwari 2021). In the absence of recognized gold standard for the assessment of POCD, neuropsychological tests—such as DSM‐5, Confusion Assessment Method for the Intensive Care Unit (CAM‐ICU), 3‐minute Diagnostic Interview for Delirium using the confusion assessment method (3D‐CAM), Montreal Cognitive Assessment (MoCA), and Addenbrooke's Cognitive Exam III (ACE‐III), Wechsler Memory Scale—are commonly used to evaluate cognitive decline (Ely et al. 2001; Kong, Xu, and Wang 2022; Marcantonio et al. 2014; Senda et al. 2020). Additionally, the advent of neurophysiological examinations, magnetic resonance imaging, tau protein, and artificial intelligence (AI) has facilitated the determination of the occurrence of POCD (Boord et al. 2024; Lee et al. 2023; Mao et al. 2024; Park et al. 2024; Reese et al. 2024; Sorby‐Adams et al. 2024).

### 4.2 Summary of Main Findings

From a bibliometrics perspective, the growing number of studies on neuroinflammation‐induced POCD indicate an increased interest in this field. The qualitative and quantitative analyses results using VOSviewer and CiteSpace indicate that the number of research papers related to neuroinflammation‐induced POCD has exhibited a consistent and upward trend over the past 12 years. Notably, since 2021, there has been a remarkable surge in literature volume, indicating an increased recognition of the vital role of neuroinflammation in POCD development.

China ranked first in neuroinflammation‐induced POCD studies, highlighting its rapid development and strong scientific research potential. However, China has to enhance research quality, as evidenced by the considerable disparity in average citation rates between China and top ranked nations such as the USA. Additionally, given the limited extent of cooperation among countries/regions, it is imperative to enhance cross‐regional and transnational collaboration and dismantle the academic barriers to advance research even further. Furthermore, institutional and cultural differences must be addressed with concerted efforts. Also, there is an urgent need to develop a common understanding and consensus to guarantee successful cooperation.

Analysis of the higher‐impact journals showed that *Journal of Neuroinflammation* had the highest volume and of citations relevant published literature. Notably, this journal explored the connections between the immune and nervous systems, as well as how they are affected by factors such as age, injury, illness, or degenerative conditions. Consequently, this journal holds significant influence in the field of neuroinflammation‐induced POCD, making it a focal point for researchers interested in this area.

Niccolò Terrando was the most frequently co‐cited author and most co‐cited reference was the study by Hovens et al. from Yale University published in *Brain Behavior and Immunity* in 2014 (Hovens et al. 2014). Their findings have revealed temporal alterations in behavior, neuroinflammation, and the BDNF pathway characterize young adult rats after surgery. Notably, the disparities in their temporal progression and correlation with plasma IL‐6 indicate that sickness behavior and cognitive decline are distinct features of POCD.

### 4.3 Overview of Knowledge Maps and Research Hotspots

Based on our analyses of research themes and keywords, four prominent research trends and hotspots were identified through the network analysis of neuroinflammation‐induced POCD: anesthesia, surgery, dexmedetomidine, NLRP3 inflammasome and mechanism of neuroinflammation‐induced POCD.

#### 4.3.1 Anesthesia

The neuroinflammation induced by anesthesia and surgical procedures is a prevalent cause of POCD. Numerous studies have investigated and demonstrated the impact of inhaled anesthetics on POCD (Zeng et al. 2023; Zhao et al. 2021). Numerous studies have demonstrated that sevoflurane can enhance inflammatory response of the nervous system and induce apoptosis, which is closely associated with the development of POCD (Wu et al. 2022; Zhao et al. 2021). Previously, we identified that sevoflurane‐induced neurotoxicity and neuroinflammation exacerbates POCD (Chen et al. 2022). Additionally, several high‐quality randomized controlled trials (RCTs) have demonstrated that sevoflurane anesthesia elevates the concentration of S100‐β protein, TNF‐α, and IL‐6 in the plasma of patients (Qiao et al. 2015). Conversely, propofol was associated with a lower occurrence of POCD compared to both sevoflurane and isoflurane (Geng, Wu, and Zhang 2017). Additionally, the occurrence of POCD can be mitigated by optimizing the depth of anesthesia—as monitored by tools such as the bispectral index (BIS)—and through the administration of certain drugs, such as lidocaine (Chen et al. 2015; Quan et al. 2019).

#### 4.3.2 Surgery

Surgery is another significant factor contributing to POCD. Through dynamic evolution analysis of neuroinflammation‐induced POCD, we have revealed that in the recent years, clinical studies on neuroinflammation‐induced POCD following cardiac, non‐cardiac, and major abdominal surgical procedures have emerged as the primary research focus. A recent meta‐analysis has revealed that patients undergoing cardiac and non‐cardiac surgeries exhibit poor prognosis and an augmented risk of premature death when they experience POCD within 30 days (Suraarunsumrit et al. 2024). Consequently, significant and crucial for anesthesiologists to precisely and accurately identify the incidence of POCD associated with various types of surgeries.

#### 4.3.3 Dexmedetomidine

Dexmedetomidine is a highly selective α_2_‐adrenoceptor agonist. Numerous studies have confirmed that it has anti‐inflammatory and anti‐apoptosis effects. Dexmedetomidine reduces the expression of IL‐Ιβ, IL‐6, and TNF‐α, as well as inhibits the activation of astrocytes and microglia in the hippocampus (Zhu et al. 2016). Additionally, dexmedetomidine exhibits neuroprotective abilities against POCD through the mechanisms, the miR‐381/EGR1/p53 signaling pathway, NLRP3 inflammasome signaling pathway, HDAC2/HIF‐1α/PFKFB3 axis, and MicroRNA‐103a‐3p/VAMP1 axis (Cho et al. 2022; Liu et al. 2022; Wang, Zhang, and Cai 2021; Wu, Wang, Shi, and Li 2022). Clinically, dexmedetomidine demonstrates a potential to mitigate the occurrence of POCD in short‐ and long‐term periods, especially in cardiac surgery (Qin et al. 2021; Subramaniam et al. 2019; Yu et al. 2022; Zeng et al. 2023). However, conflicting findings suggest that intraoperative infusion of dexmedetomidine may not effectively prevent POD in elderly patients (Turan et al. 2020). Therefore, we speculate that the neuroprotective efficacy of dexmedetomidine is dependent on the mode and dosage of administration. However, this claim warrants validation through further investigations encompassing both fundamental and clinical studies.

#### 4.3.4 NLRP3 Inflammasome

The NLRP3 inflammasome is a cellular complex, consisting of proteins containing the NOD‐, LRR‐, and pyrin domains. Notably, it is implicated in the inflammatory pathway of the innate immune system (Shao et al. 2020). The activation of the NLRP3 inflammasome is typically initiated by factors such as pathogenic infections, cellular damage, or environmental stimuli. Upon its activation, NLRP3 facilitates the maturation and release of IL‐1β and IL‐18, thereby increasing the inflammatory response (Tork et al. 2024). This complex is predominantly expressed in neural glial cells, like microglias and astrocytes, and its activation induces these cells to secrete pro‐inflammatory cytokines, further intensifying neuroinflammation (Tork et al. 2024; Xu et al. 2023; Zhou et al. 2023). Numerous studies show that the CNS inflammatory response induced by the activation of NLRP3 inflammasome plays an important role in the occurrence and development of neurodegeneration (Barnett et al. 2023; Tan et al. 2021). The targeted inhibition of NLRP3 activation or its downstream signaling pathways represents a potential therapeutic strategy for addressing neuroinflammation‐induced POCD (Sun et al. 2022; Zhao et al. 2021).

#### 4.3.5 Mechanism of Neuroinflammation‐Induced POCD

In the studies involving the underlying mechanism of neuroinflammation‐induced POCD, two domains have garnered attracted significant attention: the BBB and activation of microglia. Consequently, the targeted prevention and treatment of BBB, as well as microglia activation represent a vital area of research focus within the neuroinflammation‐induced POCD domain.

The BBB is a crucial component in safeguarding the brain against immune‐mediated injuries. Notably, BBB is highly specialized endothelial membrane with the capacity to develop impermeable barriers, thereby effectively restricting the infiltration of harmful molecules and immune cells into CNS (Ransohoff and Engelhardt 2012). An increasing body of evidence indicates that BBB is disrupted in POCD in aged rodent models exposed to various surgeries or isolated anesthesia, potentially involving diverse molecular mechanisms (Cao et al. 2018; Hu et al. 2025; Ni et al. 2018; Zhang et al. 2016). The disruption of BBB is predominantly age‐dependent, indicating increased BBB permeability, which acts as a risk factor for POCD (Yang et al. 2017; Zhu, Liu, and Fang 2018).

The underlying mechanisms of neuroinflammation‐induced POCD, particularly microglia, have been extensively studied. The microglia play an important role in maintaining cerebral homeostasis and normal brain function, thereby facilitating neurodevelopmental processes (Askew et al. 2017; Degos et al. 2013; Liu et al. 2025; Yang et al. 2024). Microglia are highly responsive to external stimuli and can be activated in response minor changes within CNS. After activation, microglia undergo polarization into two distinct types: M1 pro‐inflammatory phenotype and M2 anti‐inflammatory phenotype (Gordon and Martinez 2010; Long et al. 2024). The M1 pro‐inflammatory phenotype releases proinflammatory cell mediators, aggravating inflammatory response, whereas the M2 anti‐inflammatory phenotype secretes anti‐inflammatory factors and neurotrophies to facilitate damage repair; therefore, the dual characteristics of microglia and M1/M2 polarization play an important role in the development of various diseases (Long et al. 2024; Yang, Xu, Qian, and Xiao 2017). The surgical and anesthetic‐induced noxious stimulation result in increased levels of inflammatory factors in the surrounding tissues, alterations in the microglia morphology, and production of various inflammatory factors such as TNF‐α, IL‐1, and IL‐6.(Norden and Godbout 2013) Previous studies have demonstrated a close association between cognitive impairment elevated levels of the inflammatory factor and activated microglia in mice models (Choi et al. 2024; Cibelli et al. 2010; Li et al. 2023). Inhibition of microglial activity reduces levels of proinflammatory cytokines such as TNFα and IL‐1β, along with MCP‐1 chemokine (Feng et al. 2017). Studies also show that the involvement of microglia in the inflammatory response is also mediated by the nuclear transcription factor κB (NF‐κB) signaling pathway and Toll‐like receptor 4 (TLR‐4). This process encompasses a various inflammatory factors, including IL‐1β, IL‐6, nitric oxide synthase 2 (NOS2), reactive oxygen species (ROS), tumor necrosis factor‐alpha (TNF‐α), cyclooxygenase‐2 (COX‐2), and acute phase proteins such as pentraxin‐3 (Dutta et al. 2023; Ma et al. 2022; Yamanaka et al. 2017). Collectively, these findings demonstrate that microglia plays a significant role in the development of neuroinflammation‐induced POCD.

### 4.4 Future Frontiers

Results from the analysis of the dynamic evolution of neuroinflammation‐induced POCD, and the burst test, facilitated the identification of several future trends in this field, including studies involving POD, PND, apoptosis, and epigenetic modifications.

#### 4.4.1 POD and PND

Recently, the concepts of POD and PND have garnered increased attention and research especially with the issue of the global aging population. The mechanism underlying the development and progression of PND remains elusive within the basic research domain. However, following the integration of the concept of PND, neuroscientists and anesthesiologists have been aiming to elucidate its underlying mechanisms. It can be conclusively stated that mechanisms involving BBB, synaptic plasticity impairment, oxidative stress, neuroimmune, and neural circuit homeostasis play significant roles in this context (Cheng et al. 2022; Wang et al. 2023; Wei et al. 2024; Xu et al. 2023; Zhang et al. 2024). While research on PND is majorly basic, the clinical research of POD is more extensive. In recent years, POD‐related clinical research has emerged as a hot topic in anesthesiology, encompassing two significant aspects: POD risk factors and prevention of POD (Li et al. 2022; Swarbrick and Partridge 2022; Zarour et al. 2024). Multiple high‐quality RCTs have identified that factors such as dexmedetomidine are capable of preventing POD (Deiner et al. 2017; Ma et al. 2024; Momeni et al. 2021; Sieber et al. 2019; Subramaniam et al. 2019; van Norden et al. 2021). In the foreseeable future, the prevention of POD and PND from both basic and clinical perspectives will remain a hot research topic within the field of anesthesiology.

#### 4.4.2 Apoptosis

Apoptosis is a programmed cell death process; it involves self‐annihilation of cells via the activation of endogenous and exogenous signals (Bredesen 1995; Newton et al. 2024). Apoptotic balance in physiological and pathological conditions is vital for the maintenance of homeostasis (Bredesen 1995; Newton et al. 2024; Yuan and Ofengeim 2024). Given the significance of NLRP3 inflammasome and microglia activation, extensive research has been conducted. Notably, recent investigations have focused on exploring the mechanism of apoptosis causing POCD within the context of NLRP3 inflammasome and microglia, thereby emerging as another significant research topic, with numerous related publications released between 2023 and 2024 thoroughly highlighting the mechanism of POCD (Chen et al. 2024; Jin et al. 2019; Kong et al. 2024; Zhang et al. 2023; Zhou et al. 2023).

#### 4.4.3 Epigenetic Modifications

Epigenetics modifications—defined as phenomena in which the genetic composition remains unaltered but significant phenotypic changes occur—was used to examine various aspects of development, including DNA methylation, histone modifications, and non‐coding RNA (Allis and Jenuwein 2016; McEwen et al. 2015). Epigenetic modifications have thrived in the field of neuroscience in recent years, with similar success observed in research related to POCD (Allis and Jenuwein 2016; Luo, Min, Wu, and Zuo 2020; Min et al. 2022; Wu et al. 2023; Zhou et al. 2023). In 2017, Zhu et al. demonstrated that DNA methylation of the glucocorticoid receptor is closely associated with the induction of neuroinflammation and POCD (Zhu et al. 2017). With advancements in experimental techniques, such as sequencing, epigenetic modifications provide a promising research area involving investigations on the mechanism of neuroinflammation‐induced POCD.

## 5 Limitations

This research has several limitations. First, we only conducted searches in the WoSCC database and did not include other databases such as PubMed, Embase, Scope, and Google Scholar. Although the WoSCC encompasses high‐quality academic publications, it is possible that articles published in other databases may have been overlooked, thereby limiting the scope of our results. Second, WoSCC is constantly updated leading to miss of new and potential important data. Third, data collection was limited to English original articles and reviews, resulting in linguistic bias. Given that only English‐language publications were included, the potential for diverse perspectives and insights from other languages was missed. Finally, there is a potential for underappreciation of certain high‐quality studies due to their low citation rate. Nonetheless, it is important to note that citations are influenced by multiple factors and inherently do not reflect the scholarly quality. Furthermore, due to the “Sleeping Beauty” phenomenon, some recently publications fail obtain effective citations, which potentially leads to citation bias (Kokol, Blažun Vošner, and Vermeulen 2017).

## 6 Conclusions

This study, to the best of our understanding, represents the first comprehensive assessment of the dynamic evolution of themes related to neuroinflammation‐induced POCD using bibliometric methods. Our analysis identified the following key research areas related to neuroinflammation‐induced POCD: anesthesia, surgery, dexmedetomidine, NLRP3 inflammasome, and mechanism of neuroinflammation‐induced POCD. Additionally, POD, PND, apoptosis, and epigenetic modifications were identified as future research trends and frontiers.

## Author Contributions

**Zheping Chen**: conceptualization, writing–original draft, writing–review and editing, investigation. **Zhenxiang Zuo**: writing–original draft, writing–review and editing. **Yizheng Zhang**: methodology, software, formal analysis, writing–review and editing. **Guoliang Shan**: methodology, software, formal analysis, writing–review and editing. **Le Zhang**: formal analysis. **Moxuan Gong**: formal analysis. **Yuyang Ye**: formal analysis. **Yufeng Ma**: data curation. **Yanwu Jin**: conceptualization, investigation, writing–review and editing, validation, supervision, funding acquisition.

## Conflicts of Interest

The authors declare no conflicts of interest.

### Peer Review

The peer review history for this article is available at https://publons.com/publon/10.1002/brb3.70271.

## Data Availability

The data that support the findings of this study are available from the corresponding author upon reasonable request.
